# Modified sleeve gastrectomy technique with Hemoben hemostatic powder: prevention of early postoperative bleeding

**DOI:** 10.1093/jscr/rjag334

**Published:** 2026-05-06

**Authors:** Alijon Salimovich Murodov, Jamol Kamalovich Toshpulatov, Jafar Zafarovich Juraev

**Affiliations:** Department of Surgical Diseases in Family Medicine, Tashkent State Medical University, Tashkent Almazar district, Farobii st. 2, 100109, Uzbekistan; Department of Surgical Diseases in Family Medicine, Tashkent State Medical University, Tashkent Almazar district, Farobii st. 2, 100109, Uzbekistan; Department of Surgical Diseases in Family Medicine, Tashkent State Medical University, Tashkent Almazar district, Farobii st. 2, 100109, Uzbekistan

**Keywords:** sleeve gastrectomy, hemostatic powder, staple line bleeding, bariatric surgery, surgical technique

## Abstract

Postoperative bleeding from the staple line remains a significant complication after laparoscopic sleeve gastrectomy, occurring in 0.6%–3.4% of cases. This study presents a modified surgical technique utilizing Hemoben hemostatic powder combined with omental reinforcement to prevent early bleeding complications. Between 2019 and 2022, 187 patients with morbid obesity underwent laparoscopic sleeve gastrectomy. The intervention group (*n* = 95) received the modified technique with Hemoben powder application and distal staple line reinforcement with omentum, while the control group (*n* = 92) underwent conventional oversewing. The modified technique demonstrated lower bleeding rates (1.05% vs 3.26%, *P* = .36, Fisher’s exact test; 95% confidence intervals provided), reduced operative time by 10–15 min, and decreased transfusion requirements. No reoperations for bleeding were needed in the intervention group compared to one case in controls. The technique is simple, cost-effective, and provides reliable hemostasis without prolonging surgery duration. This modified approach demonstrates promising observational results as an alternative for preventing staple line bleeding in sleeve gastrectomy procedures and requires prospective multicenter validation.

## Introduction

Laparoscopic sleeve gastrectomy (LSG) has emerged as the most commonly performed bariatric procedure worldwide, accounting for over 60% of all metabolic surgeries [1]. Despite its technical simplicity and proven efficacy, postoperative complications remain a concern, with staple line bleeding representing one of the most frequent non-septic complications, occurring in 0.6%–3.4% of cases [2, 3]. Bleeding complications not only increase morbidity but also prolong hospital stay and significantly elevate healthcare costs, with an estimated additional cost of $5261 per bleeding episode [4].

Various strategies for staple line reinforcement have been proposed, including oversewing, buttressing materials, and hemostatic clips [5, 6]. However, the optimal method remains controversial, with studies showing variable results regarding effectiveness and cost-efficiency [7]. Traditional oversewing techniques, while widely used, add 10–15 min to operative time and require meticulous surgical technique [8].

The development of hemostatic powders has opened new possibilities for managing surgical bleeding. These agents act through multiple mechanisms, including absorption of fluid, concentration of clotting factors, and mechanical tamponade [9]. We present a modified sleeve gastrectomy technique utilizing Hemoben hemostatic powder (Uzbekistan) combined with selective omental reinforcement to achieve effective hemostasis while simplifying the surgical procedure.

## Methods

### Study design and patient selection

This is a comparative cohort study with retrospective control group and prospective intervention group, presented primarily as a surgical technique description with supportive observational data. A total of 187 patients with morbid obesity were comprehensively diagnosed and treated.

The control group (*n* = 92) consisted of patients treated between January 2019 and June 2021 who underwent conventional sleeve gastrectomy with complete staple line oversewing—a retrospective cohort. The intervention group (*n* = 95) consisted of patients treated between July 2021 and December 2022 who underwent our modified sleeve gastrectomy technique with the aim of reducing early complications (staple line insufficiency and bleeding)—a prospective cohort.

All procedures were performed by the same surgical team (two experienced bariatric surgeons with >100 LSG cases each prior to study initiation), minimizing learning curve bias. During the initial study period, 32–34 Fr bougie calibration was occasionally used; however, based on observed complications (dyspepsia in mild cases, staple line insufficiency, and increased gastroesophageal reflux disease (GERD) symptoms in severe cases), we standardized to 36 Fr bougie for all subsequent cases, using smaller calibration probes only in individual circumstances.

Perioperative care evolved into a comprehensive three-phase protocol: (i) preoperative phase: patient selection, metabolic stabilization, and coagulation system normalization; (ii) intraoperative phase: standardized bougie selection and avoidance of aggressive staple line manipulation; (iii) postoperative phase: anticoagulation therapy (low-molecular-weight heparin) adjusted for body mass index (BMI), comorbidities, and metabolic status, initiated preoperatively and continued under coagulation monitoring.

We acknowledge that the sequential nature of the cohorts may introduce temporal confounding. The findings should be interpreted as preliminary observational data supporting technique feasibility rather than definitive evidence of superiority.

Inclusion criteria: BMI ≥40 kg/m^2^ or BMI ≥35 kg/m^2^ with obesity-related comorbidities, age 18–65 years, failed conservative weight loss attempts, suitable candidates for laparoscopic surgery. Exclusion criteria: previous bariatric surgery, coagulation disorders, chronic anticoagulant therapy, severe cardiopulmonary disease contraindicating surgery. All cases were consecutive within each study period.

### Definitions

Early postoperative bleeding was defined as bleeding observed within 72 h after the surgical procedure. The main cause is bleeding from the staple line. Clinical criteria included: tachycardia, decreased blood pressure, and hemoglobin level decrease ≥20 g/l compared to preoperative values.

Visualization criteria included: abdominal drain tube monitoring (if placed), endoscopy when there is risk of intraluminal bleeding in the gastric sleeve, and dynamic abdominal ultrasound (checking for free fluid in the abdominal cavity).

Indications for blood transfusion included hemodynamic instability caused by bleeding: systolic blood pressure drop below 90 mmHg, tachycardia (110–120 beats per minute), hemoglobin decrease below 70 g/l, and corresponding clinical signs such as cold sweats, dizziness, and weakness.

During our scientific research, we obtained a copyright certificate from the Intellectual Property Agency of the Republic of Uzbekistan for the software product “Early Diagnosis of Staple Line Insufficiency After Sleeve Gastrectomy in Patients with Morbid Obesity” (RDNSL-PRJ-BMO.exe), dated 13 March 2023, No. DGU 23232. Using this software product, early diagnosis of disease complications is made, and treatment measures are started as timely as possible (Table 1). This allowed for elimination of early postoperative complications in patients before they reached the level of generalized problems in the body.

**Table 1:** Early diagnosis scoring system for staple line insufficiency after sleeve gastrectomy in patients with morbid obesity.

Symptoms	Presence
1. Sudden onset of severe abdominal pain	Absent 0/Questionable 1/Present 2
2. Presence of tachycardia	Absent 0/Moderate 1/Severe 2
3. Presence of leukocytosis	Absent 0/Moderate 1/Severe 2
4. Elevated temperature	Normal 0/Subfebrile 1/High 2
5. Pain and discomfort in epigastric area	Absent 0/Moderate 1/Severe 2
6. Presence of bloating	Absent 0/Moderate 1/Severe 2
7. Presence of free fluid in abdominal cavity	Absent 0/Trace 1/Localized collection 2
8. X-ray or CT signs of contrast extravasation outside the organ (extraorgan)	Absent 0/Questionable 1/Present 2

Validated scoring system for early detection of staple line leak. Each of the 8 symptoms is scored 0, 1, or 2 points (total range 0–16). Scores 0–5 indicate no leak; 6–10 indicate suspected leak requiring monitoring; 11–16 indicate absolute indication for relaparoscopy.

Score interpretation:

- 0–5 points—the patient has no signs of staple line insufficiency
- 6–10 points—there is suspicion of staple line insufficiency and dynamic monitoring of the patient’s condition in hospital conditions is indicated
- 11–16 points—there is an absolute indication for relaparoscopy for staple line insufficiency

### Postoperative monitoring protocol

All patients in both groups underwent standardized postoperative surveillance based on three monitoring domains:

Vital signs monitoring: blood pressure, heart rate, respiratory rate, SpO₂, temperature, urine output, and pain intensity were recorded every 4 h for the first 24 h, then every 8 h until discharge.

Laboratory monitoring: complete blood count (including hemoglobin and hematocrit) was obtained once within the first 12 h postoperatively if the procedure was uncomplicated. If tachycardia developed with hemodynamic deterioration or hemorrhagic drain output was observed, hemoglobin was repeated immediately.

Drain placement: routine drain placement was not standard practice at our institution. Drains were placed selectively in cases of: intraoperative difficulty (prolonged procedures, adhesions, difficult staple line management), suspected bleeding, or presence of super obesity with significant comorbidities (e.g. arterial hypertension).

Indications for endoscopy: upper gastrointestinal endoscopy was performed when intraluminal bleeding was suspected: “coffee ground” emesis, melena, or progressive hemoglobin decline (>30 g/l) with stable external drainage. Endoscopy was also performed when staple line insufficiency was suspected: sudden severe epigastric pain with tachycardia and fever, for both diagnostic and therapeutic purposes.

Blood transfusion criteria: transfusion was indicated for hemodynamic instability due to bleeding: systolic blood pressure <90 mmHg, tachycardia (110–120 bpm), hemoglobin <70 g/l, or corresponding clinical signs (cold sweats, dizziness, weakness).

Indications for relaparoscopy for bleeding: surgical reintervention was indicated when: hemodynamic instability persisted despite resuscitation (hypotension, tachycardia, shock signs, or vasopressor requirement); persistent or progressive hemoglobin decline occurred despite transfusion; high-volume drain output was observed (>100–150 ml/h or >500 ml total); or imaging (ultrasound or computed tomography (CT)) showed hemoperitoneum with clinical deterioration. Conservative measures were attempted first (fluid resuscitation, transfusion), and relaparoscopy was performed without delay if the patient’s condition worsened or failed to stabilize.

Indications for relaparoscopy for staple line insufficiency: decision was based on clinical signs, overall patient condition, degree of sepsis, and imaging findings. Relaparoscopy was indicated for: hemodynamic instability or septic shock (tachycardia, hypotension, vasopressor requirement); generalized peritonitis (severe abdominal pain, peritoneal signs, endointoxication, fever); or CT evidence of contrast extravasation from the gastric sleeve with intra-abdominal fluid collection (localized or diffuse), requiring source control and adequate drainage.

### Statistical analysis

Quantitative variables were described as arithmetic mean ± standard deviation. Qualitative variables were presented as absolute and relative frequencies (percentages). Values at the α < 0.05 level were considered statistically significant. *P*-values were calculated two-sided. Statistical methods used included: paired Student’s t-test, Fisher’s exact test for categorical variables, and Mann–Whitney U-test for continuous variables. Pearson correlation coefficient was used for interval and nominal scale variables, Spearman’s rank correlation for ordinal scales. Multifactorial logistic regression analysis was used to study endpoint predictors. Kaplan–Meier analysis was used to study freedom from reoperation rates. Receiver Operating Characteristic (ROC) curve analysis was performed for endpoint predictors. Calculations were performed using Microsoft Excel and SPSS 22.0. 95% confidence intervals (95% CI) were calculated for bleeding and staple line insufficiency rates using the Wilson score method.

## Surgical technique

### Patient selection and preparation

Patients with BMI ≥35 kg/m^2^ with obesity-related comorbidities or BMI ≥40 kg/m^2^ were selected for LSG following standard preoperative evaluation. All procedures were performed under general anesthesia with the patient in supine reverse Trendelenburg position.

### Standard LSG procedure

A five-trocar technique was employed. After gastrocolic ligament division and greater curvature mobilization from 4 to 6 cm proximal to the pylorus to the angle of His (Fig. 3), a 36-Fr bougie was inserted along the lesser curvature. To reduce the frequency of postoperative complications, principles of safe gastric calibration were strictly followed, avoiding excessive narrowing of the tube lumen, especially in the antral and cardial sections. Small-caliber calibration probes (smaller than 36 Fr) were not used, especially in patients with risk factors for gastroesophageal reflux. Gastric resection was performed using endoscopic linear staplers (Echelon, Ethicon Endo-Surgery) with sequential firing strictly along the bougie. The first cartridge used was green (for thicker antral tissue), and subsequent cartridges were blue (for thinner gastric tissue).

### Modified hemostatic technique

Following completion of gastric transection, our modified approach consists of three key components:

Hemoben powder application: immediately after staple line completion, Hemoben hemostatic powder is uniformly applied along the entire staple line length. A typical amount of 1.0–1.5 g of powder is used per case, depending on staple line length (average 15–20 cm).

**Figure 1:**
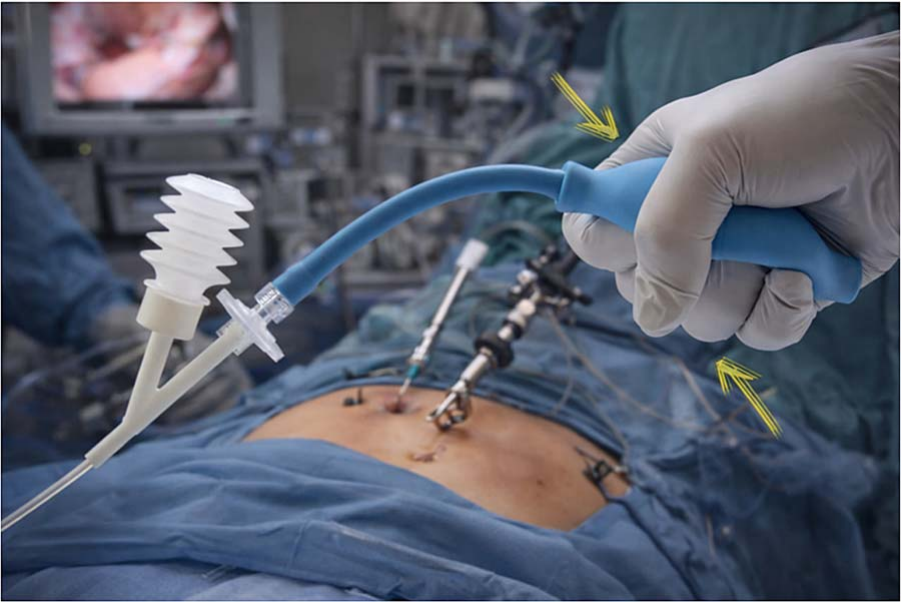
Laparoscopic powder applicator device for hemostatic powder delivery during sleeve gastrectomy. The applicator tip is inserted through a standard trocar to enable uniform powder distribution along the staple line under reduced pneumoperitoneum pressure (6 mmHg).

The powder is delivered using a standard laparoscopic powder insufflator-type applicator (Fig. 1). The applicator consists of a cannula with a flexible tip that passes through a standard trocar. Similar commercially available devices include the SURGICEL™ Endoscopic Applicator (Ethicon), HEMOBLAST Bellows Laparoscopic Applicator (Biom’up), and FlexiTip™ Spray Applicator (BD).

Technique for ensuring uniform layer thickness: To achieve consistent powder distribution, pneumoperitoneum pressure is reduced from the standard 12–15 to 6 mmHg before powder application. This pressure reduction creates optimal conditions for uniform powder deposition along the staple line and prevents powder dispersion into the peritoneal cavity. The applicator tip is maintained at a consistent distance of 2–3 cm from the staple line surface, using slow sweeping motions along the entire length under direct visualization. Complete coverage is confirmed visually by the characteristic cream-colored coating of the entire staple line (Fig. 4). No irrigation is performed for 3–5 min to allow powder activation and gel formation.

Hemoben product information:

Composition: Na-carboxymethylcellulose, viscose, oxidized cellulose, bound calcium ions.

Description: fine-dispersed powder of light cream color for topical application.

Mechanism of action: upon contact with physiological fluids (blood, lymph, bile), the implant acquires a gel-like form and adheres firmly to the wound surface. The components activate coagulation factors V, VIII, IX, X, XI, XII, accelerate the conversion of prothrombin to thrombin, and promote thrombus formation.

Biodegradation: the powder undergoes biodegradation within 14–21 days. It is non-toxic and has no cumulative effect.

Dosing: application in an even layer up to 200 μm thick. For capillary bleeding, 1.0 g of powder provides hemostasis over an area of 25 to 100 cm^2^. Hemostasis time is 2–3 min with normal coagulation system.

Safety: intravascular use is not permitted. The powder is not intended for use in areas of blood accumulation or for stopping active venous and arterial bleeding.

**Figure 2:**
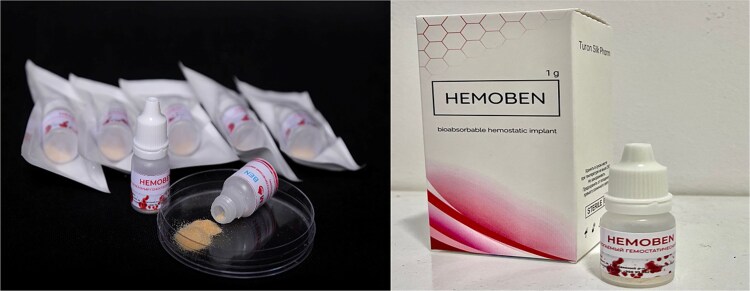
“HEMOBEN” hemostatic powder—bioabsorbable hemostatic implant (1 g) approved for clinical use in Uzbekistan.

Regulatory approval: Hemoben is approved for clinical use by the State Center for Expertise and Standardization of Medicines, Medical Devices, and Medical Equipment, Ministry of Health of the Republic of Uzbekistan (Registration No. TB/M 00539/03/22) (Fig. 2).

**Figure 3:**
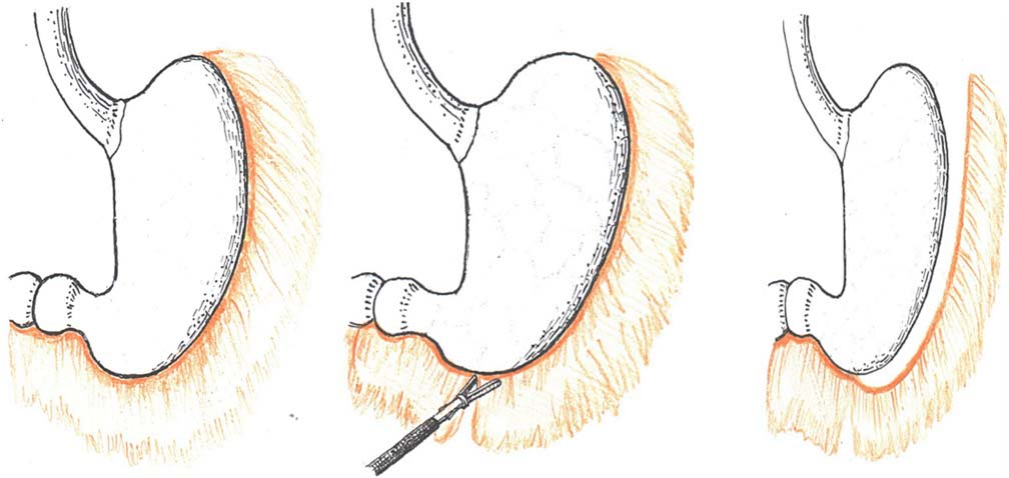
Sleeve gastrectomy—greater curvature mobilization from 4 to 6 cm proximal to the pylorus to the angle of His.

**Figure 4:**
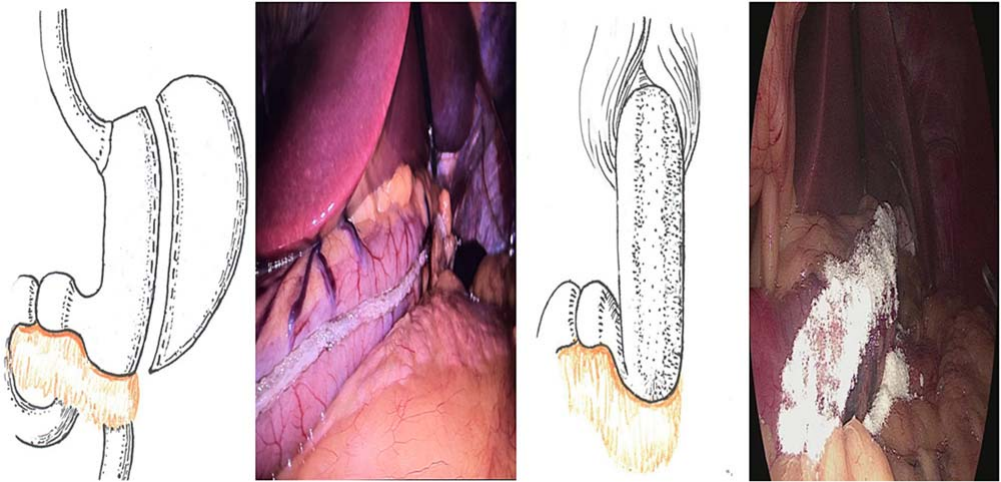
Application of hemostatic “Hemoben” powder to the gastric sleeve staple line during LSG. Note the characteristic cream-colored coating indicating complete coverage.

International availability: currently, Hemoben is commercially available in Uzbekistan and selected Central Asian countries. International readers may consider similar hemostatic powder products approved in their jurisdictions, such as SURGICEL™ Powder (Ethicon/J&J), Arista™ AH (BD), PerClot (Baxter), or 4DryField (PlantTec Medical), which share similar mechanisms of action based on polysaccharide-mediated hemostasis through rapid fluid absorption and concentration of clotting factors. The principles of our technique may be applicable with these alternative products, though specific efficacy may vary and institutional protocols should be followed.

**Figure 5:**
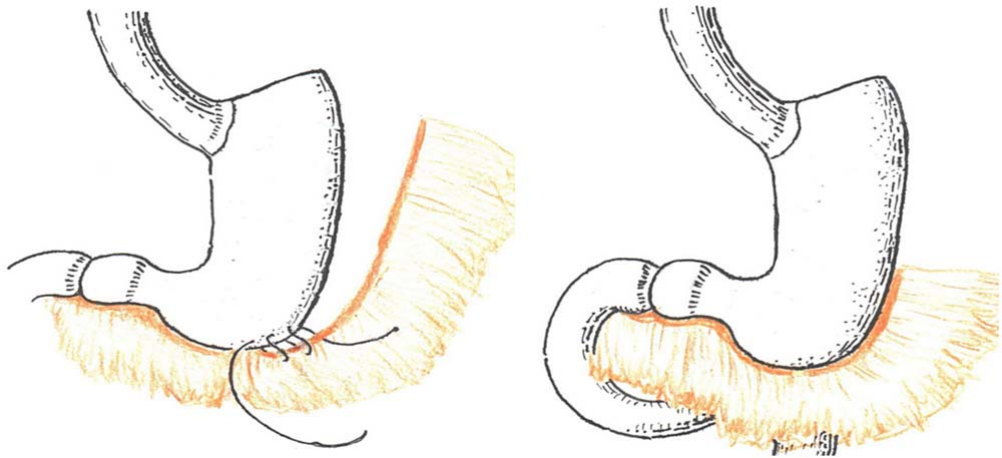
Reinforcement of the distal gastric sleeve staple line with omentum using additional sero-serosal sutures. Left: omental flap positioning; Right: completed reinforcement.

Distal reinforcement: the distal 4–5 cm of the gastric sleeve at the antral region is reinforced by wrapping a pedicled omental flap around the staple line and securing it with 2–3 interrupted 3–0 absorbable sutures (Fig. 5). This technique prevents torsion and acute deformation of the distal sleeve while providing additional hemostasis in this high-risk area. Additional reinforcement of the distal end of the gastric sleeve with a portion of omentum significantly reduces the risk of staple line insufficiency and bleeding complications in the antral zone.

Proximal fixation: the proximal gastric sleeve is fixed to the left diaphragmatic crus with a single interrupted suture to restore the angle of His and prevent fundal expansion.

No continuous oversewing of the staple line is performed, distinguishing this technique from conventional methods.

### Technical advantages

The modified technique offers several advantages: (i) simplified surgical approach eliminating the need for complete staple line oversewing, (ii) reduced operative time by 10–15 min compared to traditional methods, (iii) reliable hemostasis through dual mechanisms (powder and selective reinforcement), (iv) preservation of staple line integrity without excessive manipulation, and (v) cost-effectiveness compared to synthetic buttressing materials. When using the proposed technique, the risk of early bleeding after LSG is much lower compared to conventional methods. Dangerous bleeding or repeat interventions are virtually absent, which is associated with effective hemostasis and reduced traumatization of the gastric sleeve staple line.

## Clinical outcomes

Between 2019 and 2022, 187 patients underwent LSG at our institution. The intervention group (*n* = 95) received the modified technique, while the control group (*n* = 92) underwent conventional oversewing of the entire staple line. Groups were comparable in baseline characteristics including age, BMI, and comorbidities (Table 2).

**Table 2:** Baseline characteristics and perioperative outcomes.

Parameter	Intervention group (*n* = 95)	Control group (*n* = 92)	*P*-value
Age (years), mean ± SD	38.4 ± 10.8	39.1 ± 11.2	.68
Female, *n* (%)	67 (70.5%)	64 (69.6%)	.89
BMI (kg/m^2^), mean ± SD	44.8 ± 6.2	45.3 ± 6.7	.58
Operative time (min), mean ± SD	78 ± 12	92 ± 15	<.01
Hospital stay (days), mean ± SD	3.2 ± 0.8	3.6 ± 1.2	.08

Comparison of demographic data, body mass index, operative time, and hospital stay between the intervention group (modified technique with Hemoben powder) and control group (conventional oversewing).

Early postoperative complications are summarized in Table 3. Early postoperative bleeding requiring intervention occurred in one patient (1.05%; 95% CI: 0.03%–5.71%) in the intervention group versus three patients (3.26%; 95% CI: 0.68%–9.22%) in the control group (*P* = .36, Fisher’s exact test). While this difference did not reach conventional statistical significance due to the low event rate, it represents a clinically meaningful trend toward reduced bleeding. All bleeding episodes in the control group occurred within 24–48 h postoperatively, with one case requiring reoperation. The intervention group patient was managed conservatively with endoscopic observation and blood transfusion.

**Table 3:** Early postoperative complications.

Complication	Intervention group (*n* = 95)	Control group (*n* = 92)	*P*-value^a^
Staple line bleeding, *n* (%; 95% CI)	1 (1.05%; 0.03%–5.71%)	3 (3.26%; 0.68%–9.22%)	.36
Reoperation for bleeding, *n* (%)	0 (0%)	1 (1.09%)	.49
Blood transfusion required, *n* (%)	1 (1.05%)	4 (4.35%)	.21
Staple line leak, *n* (%; 95% CI)	0 (0%; 0%–3.8%)	2 (2.17%; 0.27%–7.59%)	.24
30-day mortality, *n* (%)	0 (0%)	0 (0%)	–

Comparison of early surgical complications between study groups, including staple line bleeding, need for reoperation, blood transfusion requirements, staple line leaks, and 30-day mortality rates. 95% confidence intervals and exact *P*-values (Fisher’s exact test) are provided for rare event comparisons.

^a^Fisher’s exact test

Mean operative time was significantly shorter in the intervention group (78 ± 12 vs 92 ± 15 min, *P* < .01). Blood transfusion was required in one patient (1.05%) in the intervention group compared to four patients (4.35%) in controls (*P* = .21, Fisher’s exact test). Hospital length of stay was comparable between groups (3.2 ± 0.8 vs 3.6 ± 1.2 days, *P* > .05), except for patients who developed bleeding complications.

At 6-month follow-up, no delayed bleeding events were observed in either group. No staple line leaks occurred in the intervention group (0%; 95% CI: 0%–3.8%) compared to two leaks (2.17%; 95% CI: 0.27%–7.59%) in the control group (*P* = .24, Fisher’s exact test). Given the small number of events, these findings should be interpreted as preliminary observational data supporting technique feasibility rather than definitive evidence of superiority. Excess weight loss was similar between groups (64.3% ± 8.2% vs 62.1% ± 7.9%, *P* > .05).

## Discussion

Our modified technique addresses several limitations of conventional staple line management. First, the application of Hemoben powder provides immediate surface hemostasis through rapid fluid absorption and concentration of clotting factors at the staple line [10]. Recent studies have demonstrated the efficacy of hemostatic powders in reducing perioperative bleeding, though their role in bariatric surgery remains under investigation [11].

Second, selective reinforcement of the distal staple line with omentum targets the most vulnerable area for both bleeding and leakage while avoiding unnecessary manipulation of the entire staple line [12]. This approach aligns with recent evidence suggesting that universal staple line reinforcement may not be necessary in all patients [13, 14].

The significant reduction in operative time is clinically meaningful, as prolonged surgical duration is associated with increased complications in bariatric surgery [15]. By eliminating complete staple line oversewing, our technique simplifies the procedure while maintaining safety.

### Cost analysis

The economic efficiency of this technique was evaluated using the cost–benefit analysis method based on direct medical costs at our institution.

Costs included:

- Operative time savings: valued at 150 000 UZS per 10 min based on operating room utilization costs (reduced by 10–15 min per case)
- Reduced hemostatic medication use: average savings of 400 000 UZS per case
- Decreased blood transfusion requirements: 800 000 UZS per unit avoided
- Reduced reintervention rates: estimated at 15 000 000 UZS per reoperation

Costs excluded: indirect costs, long-term follow-up expenses, and patient productivity losses were not calculated.

Based on these calculations, cost savings of ~1 950 000 UZS (~176 USD at average exchange rate of 11 050 UZS per 1 USD for 2022 based on Central Bank of Uzbekistan data) per patient were achieved through reduced complex therapy utilization and decreased need for additional interventions.

Important limitation: these estimates are specific to our healthcare setting in Uzbekistan and should not be directly extrapolated to other healthcare systems with different cost structures. Local cost-effectiveness analyses would be required for application in other settings.

Study limitations include its comparative cohort design with retrospective control, single-center experience, and sequential cohort design that may introduce temporal confounding. Additionally, the optimal thickness of powder application and long-term outcomes beyond 6 months require further investigation. The small number of events limits statistical power for detecting differences in rare complications, and reported differences should be interpreted as trends rather than definitive evidence.

## Conclusion

The modified sleeve gastrectomy technique utilizing Hemoben hemostatic powder with selective omental reinforcement demonstrates promising observational results as a simplified approach to preventing early postoperative bleeding. This method appears to reduce operative time while maintaining acceptable hemostatic control and safety profile. Given the limitations of the comparative cohort design, small number of events, and single-center experience, these preliminary findings require validation through prospective multicenter randomized studies before this technique can be established as a standard practice option.
